# Emerging Role of Cancer-Associated Fibroblasts-Derived Exosomes in Tumorigenesis

**DOI:** 10.3389/fimmu.2021.795372

**Published:** 2022-01-04

**Authors:** Lushan Peng, Dan Wang, Yingying Han, Tao Huang, Xiaoyun He, Junpu Wang, Chunlin Ou

**Affiliations:** ^1^ Department of Pathology, Xiangya Hospital, Central South University, Changsha, China; ^2^ Department of Ultrasound Imaging, Xiangya Hospital, Central South University, Changsha, China; ^3^ Department of Pathology, School of Basic Medicine, Central South University, Changsha, China; ^4^ Key Laboratory of Hunan Province in Neurodegenerative Disorders, Xiangya Hospital, Central South University, Changsha, China; ^5^ National Clinical Research Center for Geriatric Disorders, Xiangya Hospital, Central South University, Changsha, China

**Keywords:** cancer-associated fibroblasts, exosomes, metastasis, immune response, biomarkers, therapy

## Abstract

Cancer-associated fibroblasts (CAFs) are the most important component of the stromal cell population in the tumor microenvironment and play an irreplaceable role in oncogenesis and cancer progression. Exosomes, a class of small extracellular vesicles, can transfer biological information (e.g., proteins, nucleic acids, and metabolites as messengers) from secreting cells to target recipient cells, thereby affecting the progression of human diseases, including cancers. Recent studies revealed that CAF-derived exosomes play a crucial part in tumorigenesis, tumor cell proliferation, metastasis, drug resistance, and the immune response. Moreover, aberrant expression of CAF-derived exosomal noncoding RNAs and proteins strongly correlates with clinical pathological characterizations of cancer patients. Gaining deeper insight into the participation of CAF-derived exosomes in tumorigenesis may lead to novel diagnostic biomarkers and therapeutic targets in human cancers.

## Introduction

Approximately one and a half centuries ago, fibroblasts were first defined as spindle-shaped cells capable of collagen synthesis in connective tissues. Activated fibroblasts associated with cancer are called cancer-associated fibroblasts (CAFs) (1). Although CAFs are mainly transformed from normal fibroblasts (NFs), some can also arise from bone marrow–derived mesenchymal stem cells or as a result of conversion of adipocytes, pericytes, or endothelial cells under some rare conditions (2). The tumor microenvironment (TME) is the medium integral for tumor initiation and survival and having complex structure and functions. In recent years, aside from cancer cells, researchers focused on the role of the TME in tumorigenesis (3). Tumors occur in the TME, which includes not only tumor cells but also surrounding CAFs, mesenchymal stem cells, bone marrow–derived cells, regulatory T cells, benign endothelial cells, the extracellular matrix (ECM), and tumor-associated macrophages (4). Fibroblasts have been proven to be the most important matrix component of the TME. CAFs have been found in the tumor stroma of various cancers and identified as a special type of fibroblast (5, 6). As a key component of the TME, CAFs can promote malignant tumor behaviors mainly through the activity of some metabolic pathways and secretion of various biological factors, such as growth factors, chemokines, cytokines, and exosomes (7, 8).

In 1981, Trams et al. first described exosomes as cell-shed vesicles (with an average diameter of 30–150 nm) that can be isolated from various normal and tumor cells. In 1987, Johnstone et al. defined and named these small vesicles with membrane-based structure as exosomes (9). Exosomes are a type of extracellular vesicles (EVs) that exist in almost all kinds of body fluids, e.g., saliva, urine, and amniotic fluid (10–12). These EVs, including exosomes, were once considered cell waste products, but several studies have revealed that they can carry various cellular gene products, such as proteins, and a series of metabolites, which can be transferred to recipient cells (13). Therefore, exosomes take part in biological information exchange, thus regulating the local and distant TME, which are considered essential for tumor progression (14–17). Exosomes can be secreted by almost all cell types (18), including CAFs. The latter communicate with neighboring cells, such as tumor cells in the TME, mainly by releasing vesicles, and the most important of these vesicles are exosomes (19).

As newly studied EVs in the TME, CAF-derived exosomes have been proven to play a substantial role in tumorigenesis, including tumor cell proliferation (20), metastasis (21), drug resistance (8), and the immune response (22). Research suggests that CAF-derived exosomes regulate oncogenesis and tumor progression mainly by means of their biologically active contents (23, 24), e.g., noncoding RNAs (ncRNAs) (25), proteins (26, 27), and some metabolites.

In this review, we summarize major roles of CAF-derived exosomes in tumorigenesis and describe their molecular mechanisms of action and the relation between CAF-derived exosomal bioactive factors and clinical pathological characterizations of cancer patients. Our aim is to explore new biological markers of cancers and to outline new prospects for CAF-derived exosomes in cancer treatment.

## CAFs

### Biological Characteristics of CAFs

CAFs are a special type of fibroblast. In normal tissues, fibroblasts usually rest and are considered resting mesenchymal cells embedded in the ECM of interstitial fibers. Fibroblasts can be activated in an environment-dependent manner during wound healing, tissue inflammation, and organ fibrosis. Fibroblasts include many different subtypes that are involved in the initiation and development of different diseases. For example, papillary fibroblasts are essential for the coordination of the hair cycle and formation of hair follicles after injury. Reticulocytes mediate the early wound repair response (28). Wound repair can benefit from the proliferation of preadipocytes (29). Furthermore, myofibroblasts can be found in organs affected by fibrosis (30, 31).

CAFs can originate from NFs, bone marrow–derived mesenchymal stem cells, adipocytes, pericytes, and endothelial cells (2). The precursor fibroblasts may be resposible for the diversity of CAFs. For example, similar to myofibroblasts, CAFs from local tissue NFs are reported to highly express cytoskeletal proteins such as α-smooth muscle actin for cell contraction, whereas CAFs derived from perivascular cells are believed to be related to metastasis (32). Among these precursor fibroblasts, NFs are the main progenitors of CAFs. Several biological factors present in the TME can modulate the development of CAFs from NFs; for example, the most common mechanism of this process involves signal transduction mediated by transforming growth factor β (TGF-β) (33). Cytotoxic stimuli, such as DNA damage induced by radiation, can cause gene mutations in NFs and consequently lead to the emergence of CAFs, which is exactly the process of malignant transformation of cells (34). Moreover, cancer cell–derived exosomes have been shown to induce the conversion of NFs into CAFs by shuttling cargos, such as ncRNAs (35–39). For example, Hu et al. have demonstrated that melanoma-derived exosomal long noncoding RNA (lncRNA) Gm26809 can induce reprogramming of fibroblasts into tumor-promoting CAFs, thereby facilitating melanoma cell proliferation and migration (39).

**Figure 1:**
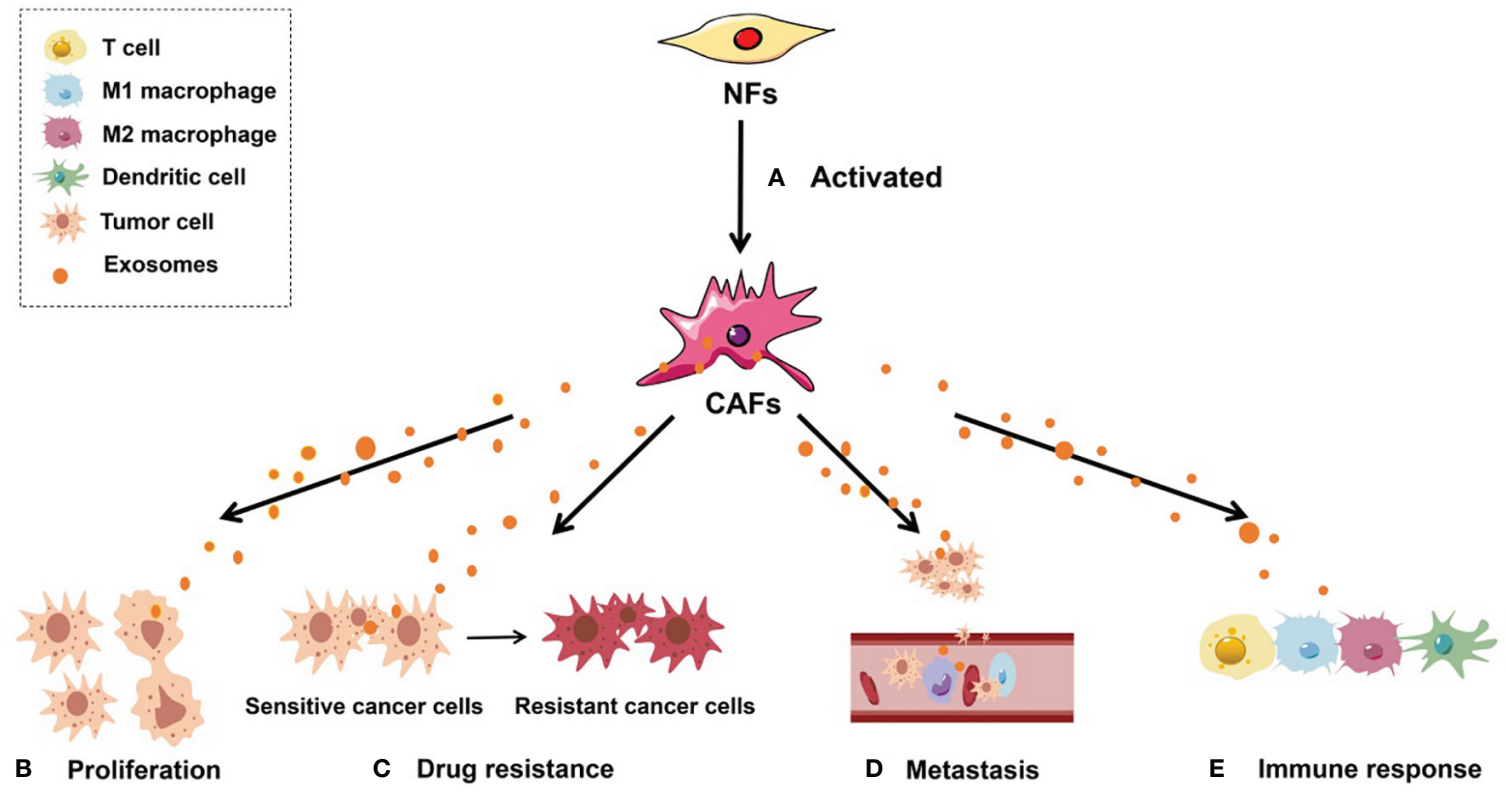
Roles of CAF-derived exosomes in tumorigenesis. **(A)** Activated NFs can be converted into CAFs. **(B)** CAF-derived exosomes can regulate tumor cell proliferation; **(C)** CAF-derived exosomes can facilitate the conversion of drug-sensitive cancer cells into drug-resistant cancer cells; **(D)** CAF-derived exosomes are able to enhance the metastatic capacity of cancer cells; **(E)** CAF-derived exosomes can induce an antitumor immune response by regulating the activity of immune cells, including T cells, M1 macrophages, M2 macrophages, and dendritic cells.

CAFs originating from NFs are unique in many aspects. The expression of “CAF markers,” such as fibroblast activation protein α and α-smooth muscle actin, distinguishes them from NFs (5). In terms of the morphological features discernible under a light microscope, CAFs have larger volume, richer cytoplasm, and a serrated nucleus, whereas under an electron microscope, one can see an abundant rough endoplasmic reticulum, free ribosomes, Golgi apparatus, and stress fibers (40). Functionally, NFs are crucial for the repair of tissue defects and participate in the protection of cells from necrosis and degeneration to various degrees (5). In contrast to NFs, activated CAFs exhibit enhanced proliferative and migratory properties and can remodel the ECM, mediate immune escape, and contribute to tumor drug resistance in the TME (41) (**Figure 1**).

### Biological Role of CAFs

As the most important component of the stromal cell population in the TME, CAFs have attracted the attention of many researchers. Numerous studies indicate that CAFs are closely related to almost all stages of tumor progression. For example, CAFs can promote malignant tumor characteristics including cancer cell proliferation, metastasis, drug resistance, and immune response by directly secreting various cytokines or chemokines (42–45) and in many other ways, such as stimulation of metastasis through the regulation of ECM remodeling and of relevant genes (46–48). Metabolic reprogramming, immune regulation, and exosome secretion are three other mechanisms by which CAFs influence the malignant behaviors of various tumors (25, 49, 50).

Metabolic reprogramming may be one of the mechanisms by which CAFs promote tumor drug resistance and has become an important way to help cancer cells acquire treatment resistance. The metabolism of CAFs is similar to that of highly proliferating cells and is based on aerobic glycolysis (51), which provides additional pyruvate and lactic acid to tumor cells and is thought to ultimately induce tumor resistance (52). Additionally, the metabolic reprogramming induced by CAFs to increase the proliferation rate of cancer cells has been verified in many studies. For example, Becker et al. have demonstrated that breast CAFs in breast cancer exhibit activated metabolism with enhanced glycolytic activity, which stimulates the growth of tumor cells (53).

CAFs can interact with various immune cells in the immune microenvironment by secreting many biological factors such as growth factors, proinflammatory cytokines, and chemokines (e.g., TGF-β and interleukin [IL]-6), thereby regulating the tumor immune response to speed up tumor progression (50, 54, 55). For instance, Harryvan et al. have found that as a cytokine capable of regulating antigen presentation, TGF-β can indirectly reduce the activation of T cells. This effect is mainly related to the role of CAFs in dendritic cells (45). Feig et al. have shown that CXCL12 from CAFs can limit the movement and/or recruitment of T cells, and plays a key role in the immune resistance of pancreatic cancer (56). In hepatocellular carcinoma (HCC), CAFs that overexpress IL-6 can induce strong immunosuppression in the TME by recruiting immunosuppressive cells such as bone marrow-derived suppressor cells and can impair the function of tumor-infiltrating T cells by upregulating suppressive immune checkpoints (27). Besides, CAFs can induce the transdifferentiation of M1 macrophages into the M2 phenotype by secreting monocyte chemotactic protein 1 and stromal cell-derived factor 1, leading to immunosuppression and increased cancer cell proliferation (57). Notably, immunomodulatory cytokines secreted by CAFs, such as IL-10, tumor necrosis factor (TNF), and interferon γ, are reported to be involved in the regulation of tumor cell immune responses by recruiting and polarizing macrophages (58, 59).

In addition, as key TME-associated mediators that have attracted much interest in recent years, exosomes secreted by CAFs are essential for the regulation of malignant tumor behaviors, mainly owing to biologically active contents of exosomes, including ncRNAs and proteins (60, 61).

## Exosomes

### Biological Characteristics of Exosomes

Exosomes are nano-microvesicles with an average diameter of 30–150 nm (62). They originate from the intracellular endosomal compartment (15), arise from the membranes of multivesicular bodies (MVBs) (18), and are formed mainly through three steps—formation of intraluminal vesicles in MVBs, transport of the MVBs to the plasma membrane, and fusion of the MVBs with the plasma membrane (63). After a successful release into the extracellular environment, exosomes can be taken up by target cells thereby transmitting biological signals between parental or distant cells. In this way, communication between tumor cells and neighboring cells, including CAFs in the TME, can be implemented successfully (64).

Recently, exosomes were investigated as suitable nanocarriers because of their biocompatibility, circulatory stability, low immunogenicity, low toxicity, and particularly small size. As an effective drug delivery platform, exosomes have aroused considerable interest regarding their usefulness for transferring anticancer drugs. For example, biocompatible tumor-cell-exocytosed exosome-biomimetic porous silicon nanoparticles have been constructed to function as a drug carrier for targeted cancer chemotherapy; when porous silicon nanoparticles are loaded with doxorubicin in the exosomal sheath, they exert anticancer action in tumor models (65).

Exosomes can be isolated by various separation and purification methods. It is imperative for researchers to find a way to obtain high-purity exosomes to advance this field of research. Therefore, the separation and purification of exosomes have always been a concern. Currently, the techniques for isolating exosomes include ultracentrifugation techniques, size-based isolation techniques, immunoaffinity capture-based Techniques, and exosome precipitation. Among them, ultracentrifugation techniques are the most popular and mainly include differential ultracentrifugation and density gradient centrifugation. Such methods as ultrafiltration, magneto-immunoprecipitation, and polyethylene glycol precipitation are often used too in size-based isolation techniques, in immunoaffinity capture–based techniques, and in exosome precipitation, respectively (66–72). Currently, there are no recognized effective extraction method for exosomes. To help researchers select the most appropriate extraction scheme, we have summarized the most commonly used Exosome isolation techniques (**Table 1**).

**Table 1:** Overview of the most popular exosome isolation techniques.

Exosome isolation techniques	Methods	Advantages	Limitations	Ref.
Ultracentrifugation techniques	Differential ultracentrifugation	Easy to use	Time consuming	(69)
		Little sample pretreatment	Requires large starting sample volumes	
		Affordability over time	Low exosome recovery	
	Density gradient centrifugation	Effective for exosomes from protein aggregates and non-membranous particles	Low exosome recovery	(70)
		Useful for separating exosomes and other EVs from body fluids		
Size-based isolation techniques	Ultrafiltration	Less time consuming	Particle deformation	(67)
		Requires no special instrumentation	Lysis of exosomes	
	Sequential filtration	Automatable	Rigid components associated with cellular debris are filtered away	(72)
		Produces intact and biologically active exosome material		
	Size exclusion chromatography	Preserves vesicle structure, integrity, and biological activity	Requires run times of several hours	(68, 71)
			Not easily scalable	
			Cannot be used for high throughput applications.	
Immunoaffinity capture-based techniques	Magneto-immunoprecipitation	Higher isolation efficiency	Protein/antigen used to capture the exosomes must be expressed on the surface of exosomes	(66, 67)
		Can handle large sample volumes		
		Preserves the activity of exosomal proteins	Specificity of the assay is limited to specificity of the antibody.	
Exosome precipitation	Polyethylene glycol precipitation	Quick	Lack of selectivity	(69)
		Simple		
		Requires little technical expertise or expensive equipment		
		Can be used for various starting volumes		

### Functions of Exosomes

EVs, including exosomes, have been thought to represent cellular waste; for example, platelet-derived EVs were once called “platelet dust” (73). In recent years, many studies have shown that EVs, particularly exosomes, are not cellular waste. They can carry proteins, a series of metabolites and cellular gene products including messengerRNAs (mRNAs) and ncRNAs such as microRNAs (miRNAs), lncRNAs, and circular RNAs (circRNAs) (74). These molecules can be transferred to recipient cells; therefore, exosomes participate in biological information exchange *in vivo*. Furthermore, exosomes carry specific proteins and nucleic acid cargo that can serve as biomarkers of many diseases, including various potentially critical illnesses (such as acute lung injury, acute kidney injury, acute myocardial injury, and sepsis), neurodegenerative diseases, tissue fibrosis, diabetes, human retroviral infections, cerebrovascular diseases, and ischemic diseases (75–82). Nonetheless, the role of exosomal contents in tumorigenesis has attracted the most attention. To date, several databases have been established to provide the latest and comprehensive information on exosomes (**Table 2**). To give an example, the ExoCarta database (www.exocarta.org) lists at least 41,860 proteins, 3,408 mRNAs, and 2,838 miRNAs that have been identified in exosomes from different species and tissues by independent studies.

**Table 2:** Exosome-related databases.

Database	Introduction	Characteristics	Website	Ref.
ExoCarta	The first comprehensive database of exosomal markers, containing 286 research results on several species, e.g., humans, rats, mice, sheep, guinea pigs, fruit flies, horses, rabbits, and cattle; data on various tissue-derived exosomal proteins, mRNA, miRNAs, and lipids and other information from organ sources are available.	ExoCarta covers the protein–protein interaction network and biological pathways with exosomal protein dynamics. Users can download the most commonly used protein data from a large number of studies. The downloaded file can be directly imported into the FunRich tool for other function enrichment analysis and correlation network analysis.	http://www.exocarta.org/	(83)
ExoRBase	A long-chain RNA-seq database of human blood exosomes. Currently, the database includes 92 blood samples, 58,330 circRNAs, 15,501 lncRNAs and 18,333 mRNAs, with annotations, expression levels and possible source tissues.	ExoRBase integrates and visualizes the RNA expression profiles based on normalized RNA-seq data spanning both normal individuals and patients with various diseases.	http://www.exorbase.org/	(84)
EVmiRNA	A miRNA database of EVs, curating and analyzing 462 miRNA expression profile datasets on EVs in 17 tissues/diseases. EVmiRNA provides several functional modules—miRNA expression profiles and the sample information about EVs from different sources; specifically expressed miRNAs in different EVs that would be helpful for biomarker identification; miRNA annotations, including miRNA expression in EVs and TCGA cancer types, miRNA pathway regulation mechanisms, and miRNA functions and literary references.	EVmiRNA provides detailed miRNA expression profiles in EVs as well as valuable and comprehensive resources, including EV samples classification (source/cancer and exosome/MV), miRNA expression profile for each sample, the most expressed miRNAs, specifically expressed miRNAs for each EV type, and miRNA functions and regulation mechanisms.	http://bioinfo.life.hust.edu.cn/EVmiRNA	(85)
EV-TRACK	A crowdsourcing knowledge base that centralizes data on EV biology and methodology and comprises methodological specifications on 3,383 EV experiments in 1,699 documents. EV-TRACK evaluates EV separation and identification-related parameters based on Minimum Experimental Requirements for EV Research.	EV-TRACK collects the original data on EV separation and characterization and increases the authenticity and repeatability of the data. For each experiment, the website explains and sort out general and specific method information to help reproduce the experiment and evaluate it.	http://www.evtrack.org/	(86)
EVpedia	A high-throughput comprehensive database of prokaryotic and eukaryotic EVs. EVpedia provides databases of prokaryotes, nonmammalian eukaryotes and mammalian vesicular mRNAs, miRNAs, and lipids.	EVpedia is an integrated and comprehensive proteome, transcriptome, and lipidome database of EVs derived from archaea, bacteria, and eukaryotes, including humans. EVpedia may serve as a useful community resource to trigger the advancement of systematic and comprehensive studies on EVs and for unveiling the fundamental roles of EVs	http://evpedia.info	(87)
Vesiclepedia	A manually curated compendium of molecular data on lipids, RNAs, and proteins identified in various classes of EVs. Currently, Vesiclepedia comprises 35,264 protein, 18,718 mRNA, 1,772 miRNA, and 342 lipid entries encompassing 341 independent studies published in the past several years.	Users can query and download EV cargo data, EV separation details, characterization methods, biophysical and molecular characteristics, and EV-METRIC according to various search criteria. This information helps biomedical scientists evaluate the quality of EV preparations and obtain the corresponding data. FunRich can help users directly analyze data.	http://www.microvesicles.org/	(88)
EMBL-EBI	A comprehensive annotation database for the functional analysis of human exosomal proteins according to Gene Ontology information.	EMBL-EBI can identify the target protein used for focus annotation and annotation of exosomal experimental methodology.	http://www.ebi.ac.uk/GOA/exosome	(89)
ExRNA Atlas	A data repository of the Extracellular RNA Communication Consortium (ERCC). This database includes small RNA sequencing and RT-qPCR-derived extracellular-RNA profiles from human and mouse biofluids.	All RNA-seq datasets are processed using version 4 of the exceRpt small RNA-seq pipeline, and ERCC-developed quality metrics are uniformly applied to these datasets.	http://exrnaatlas.org/	(90)

Exosomes can be involved in malignant tumor characteristics, including tumor cell proliferation, metastasis, drug resistance, and immune response mainly owing to their ability to carry and secrete these biologically active contents (14, 15). After studying the function of exosomes (derived docetaxel-resistant prostate cancer cells) in tumor cell proliferation and drug resistance, Corcoran et al. proposed for the first time that exosomes may be a means of transferring docetaxel-resistance between cells, which is crucial for cell communication (91). Those authors further speculated that this effect may be related to ncRNAs carried by exosomes. A series of studies have confirmed that ncRNAs carried by exosomes play an indispensable part in tumorigenesis (92). Yin et al. (93) have revealed that exosomes upregulating miR-135b-5p promoted *in vivo* growth, *in vitro* proliferation, migration, and invasion, and suppressed the apoptosis of colorectal cancer (CRC) cells. As a key component, exosomal proteins also perform vital functions in tumorigenesis. Several studies have shown that exosomal proteins, such as programmed death ligand 1 (PD-L1) and heat shock protein (HSP), participate in T-cell–mediated cellular immune responses and can activate the corresponding signaling pathways to directly influence cell apoptosis; these data make these proteins relevant for tumor resistance and tumor immunotherapy (94). Additionally, exosomal proteins help with communication between target cells through ligand–receptor interactions, is thought to be related to the participation of exosomes in tumorigenesis (95). Moreover, exosomes can carry immunosuppressive factors (96) and antigenic molecules (97) that can cause the immune system to mount an antitumor response (15) through various mechanisms, including regulation of the functions of different types of immune cells and control over antigen-dependent pathways (98).

In recent years, CAF-derived exosomes have been widely explored because of their roles as EVs in the regulation of tumorigenesis *via* secretion of various biologically active factors. On the basis of their special status as a type of small vesicle capable of information exchange in the TME, we can speculate that CAF-derived exosomes are a promising cancer-related topic and deserve deeper research. Accordingly, in the text below, we have discussed the specific molecular mechanisms of CAF-derived exosomes in tumorigenesis and their specific functions.

## The Underlying Molecular Mechanism of CAF-Derived Exosomes in Tumorigenesis

### CAF-Derived Exosomal Proteins

Proteins are an important component of CAF-derived exosomes (99). The roles of CAF-derived exosomal proteins in tumor cell proliferation, invasion, immunity, and increased metastasis have been explored extensively. The proteins seen in exosomes normally correspond to the source of exosomes, varying from different environmental conditions, with specificity. For example, antigen-presenting-cell–derived exosomes are usually rich in major histocompatibility complex molecules (96). Platelet-derived exosomes contain many hemophilia factors and integrin CD41a, while CAF-derived exosomes usually carry large amounts of death receptor ligands (such as PD-L1), and inhibitory cytokines (such as TGF-β) (100). In a study on breast cancer, the expression of PD-L1 increased after cancer cells were treated with CAF-derived exosomes, and this phenomenon may be related to the transfer of PD-L1 from the CAF-derived exosomes to cancer cells (22).

**Figure 2:**
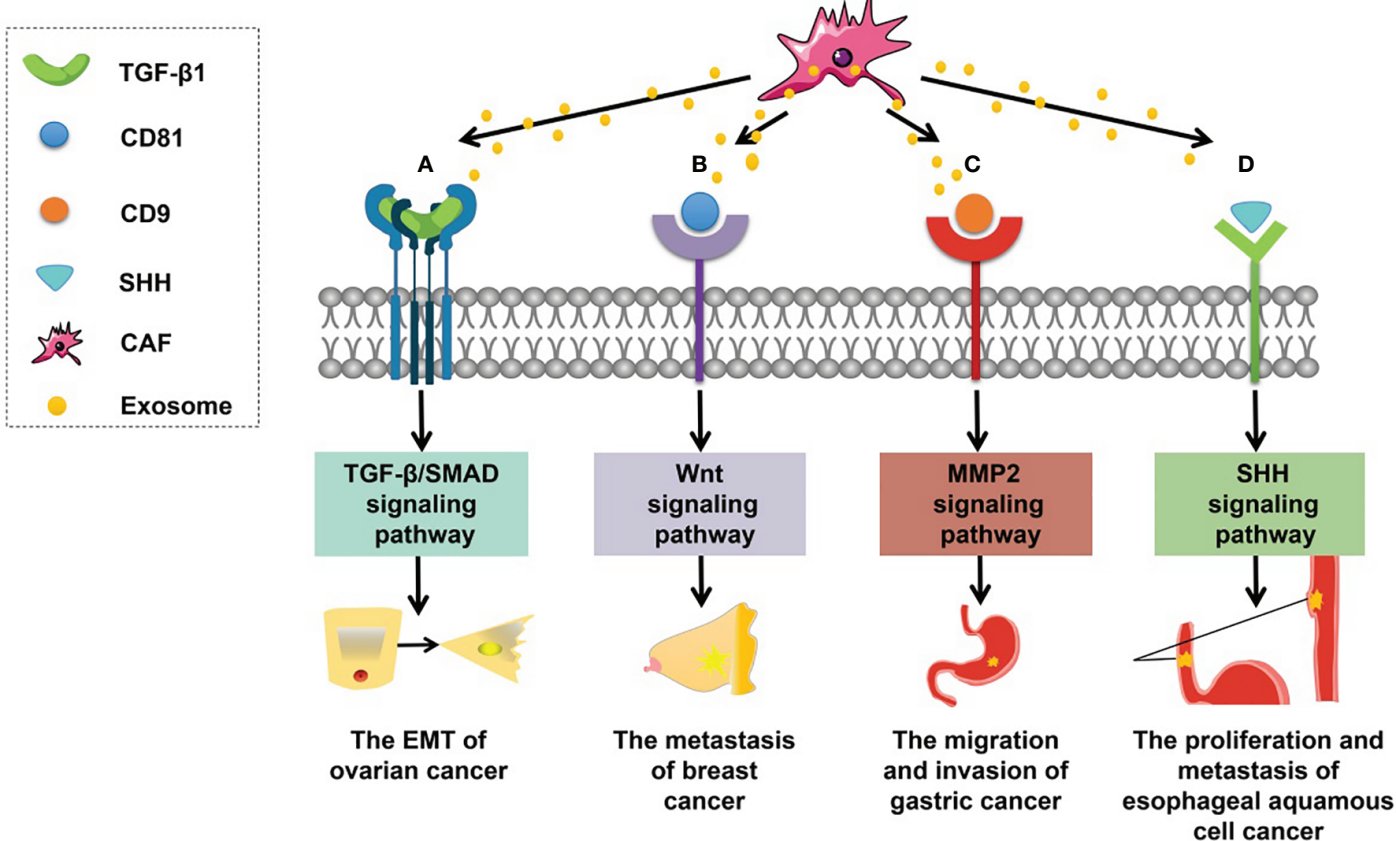
Roles of CAF-derived exosomal proteins in tumorigenesis. **(A)** CAF-derived exosomal TGF-β can activate the TGF-β–SMAD signaling pathway to promote EMT in ovarian cancer. **(B)** CAF-derived exosomal CD81 can trigger the WNT signaling cascade contributing to the metastasis of breast cancer. **(C)** CAF-derived exosomal CD9 can activate MMP2 signaling enhancing the migration and invasiveness of gastric cancer cells. **(D)** CAF-derived exosomal SHH can launch the SHH signaling pathway thus increasing the proliferation and metastasis of esophageal squamous cell cancer (ESCC) cells.

The involvement of CAF-derived exosomal proteins in tumorigenesis is mainly based on the activation of signaling pathways in recipient cells (**Figure 2**), such as the Smad and Wnt signaling pathways. For example, CAF-derived exosomal sonic hedgehog (SHH) promotes the growth and progression of esophageal squamous cell carcinoma (ESCC) by binding to the Patched protein to activate the SHH signaling pathway (60). Li et al. (101) have found that CAF-derived exosomal TGF-β1 can induce epithelial–mesenchymal transition (EMT) through the TGF-β–SMAD cascade and hence promotes the progression and metastasis of ovarian cancer. In breast cancer, after CD81-containing CAF-derived exosomes are endocytosed by cancer cells, the Wnt signaling pathway can be triggered to speed up metastasis (102). In addition, scirrhous-type gastric cancer cells can uptake CD9-positive exosomes released from CAFs; these exosomes promote cancer cell migration and invasion by activating the MMP2 signaling pathway (103).

### CAF-Derived Exosomal ncRNAs

In recent years, accumulating evidence revealed that ncRNAs play a crucial role in tumor progression. NcRNAs mainly include miRNAs, lncRNAs, and circRNAs. MiRNAs are endogenous ncRNAs with a length of 20–24 nucleotides (104, 105), which mainly binds to the 3′ untranslated region of a target mRNA to inhibit its translation and expression of the target gene, thereby affecting the initiation and progression of tumors. LncRNAs are a class of RNA molecules with sequences >200 nucleotides that lack a translated open reading frame and an encoding ability. They are also located in the nucleus or cytoplasm (106, 107). The functions of lncRNAs vary according to their subcellular location (108). When lncRNAs are located in the cytoplasm, they participate in the regulation of tumor progression by competitively adsorbing miRNAs or binding to proteins thus affecting protein modifications such as phosphorylation and can be translated into polypeptides to regulate tumorigenesis. When lncRNAs are located in the nucleus, they can bind to transcription factor–related proteins to regulate the transcriptional expression of tumor-related genes. CircRNA is a type of ncRNA (with a length of approximately 200–2000 bp; the mean length is ~500 bp) that has a closed loop structure with no free 5′ and 3′ ends and is not easily degraded by an exonuclease called RNase R (109, 110). Compared to miRNAs and lncRNAs, circRNAs are stabler, more conserved, and have cell- or tissue-specific expression patterns, indicating that they can be used as gene regulators as well as molecular diagnostic and prognostic biomarkers (111). CircRNAs are mostly expressed in the cytoplasm of eukaryotic cells, with functions similar to those of lncRNAs.

Studies suggest that ncRNAs can be secreted by CAF-derived exosomes (112). As mediators of cell communication, exosomes can transfer ncRNAs from one cell or cell line to another, thereby regulating tumorigenesis. MiRNA is a type of ncRNA that has been given the most attention. Hu et al. have reported that CAF-derived exosomes can be directly transferred to CRC cells, which significantly increases the level of miR-92a-3p, contributing to cell stemness, EMT, metastasis, and fluorouracil/oxaliplatin resistance in CRC (25). In this cancer, exosomal miR-17-5p has also been found to contribute to tumor metastasis after delivery from parental CAFs (113). In addition, Zhang et al. (114) have documented a significantly low level of miR-320a in CAF-derived exosomes by next-generation sequencing. Those authors next demonstrated that miR-320a overexpression significantly inhibits the proliferation, migration, and invasiveness of liver cancer cell lines, suggesting that augmentation of the exosomal transfer of miR-320a from stromal cells is a new strategy to suppress HCC progression. In a study on HCC, after CAF-derived exosomal miR-150-3p was transferred to HCC cells, these cancer cells were found to exhibit attenuated migration and invasiveness properties (61). Similarly, CAF-derived exosomal miR-139 inhibits the progression and metastasis of gastric cancer by repressing MMP11 expression (47). Studies on the participation of lncRNAs and circRNAs in tumorigenesis are in full swing too. A recent report showed that lncRNA H19, which was found to be enriched in CAF-derived exosomes, can act as a competing endogenous RNA (an miRNA sponge), thus taking part in tumor progression and chemoresistance (115). Likewise, CAF-derived exosomes can transfer a CRC-associated lncRNA to recipient cells to effectively induce chemotherapy resistance in CRC (116). Research on CAF-derived exosomal circRNAs is limited at present. Zhan et al. (117) have found that circHIF1A from CAF-derived exosomes can be taken up by breast cancer cells and thus plays an important role in the regulation of cancer stem cell properties by sponging miR-580-5p and therefore changing CD44 expression. In addition, exosomal circsl7a6 secreted by CAFs is reported to promote the initiation of CRC. Those authors reasonably theorized that this effect might be linked to the role of circsl7a6 in tumorigenesis mediated by sponging of tumor-related miRNAs such as members of the miR-21 and miR-200 families (118).

### CAF-Derived Exosomal Metabolites

CAF-derived exosomal metabolites are a class of small-molecule compounds that are capable of influencing malignant tumor behaviors. These metabolites include proteins, ncRNAs, lipids, amino acids, and nucleic acids that are indispensable for metabolic programming of tumors. Altered cell metabolism is a marker of cancer. Investigators have mainly focused on CAF-derived exosomal ncRNAs and proteins. To date, few studies have addressed the functions of other CAF-derived metabolites.

Supplementation of cellular nutrition through the secretion of metabolites is one of the two metabolism-regulatory mechanisms of CAF-derived exosomes affecting recipient tumor cells (119). Exosomes have been shown to stimulate the sharing of metabolites between tumor cells and CAFs, which is pivotal for tumor progression (120). In a research article on prostate cancer, Zhao et al. (119) proved that exosomes contain complete “ready-made” metabolites, including amino acids such as glutamine, threonine, serine, and valine; lipids such as palmitate and stearate; and tricarboxylic-acid cycle intermediates such as citrate, pyruvate, α-ketoglutarate, fumarate, and malate. In the case of nutritional deficiencies, these CAF-derived exosomal metabolites can be transported to cancer cells through exosomes by fibroblasts to provide fuel for the tricarboxylic acid cycle and thus to maintain the viability of tumor cells and promote tumor growth (121). The specific roles of CAF-derived metabolites in oncogenesis and progression need to be determined. Nevertheless, metabolic disorders have been observed in tumors. CAF-derived metabolites may be related to tumor metabolism, and may have the ability to regulate tumor biological behaviors, which have the potential to become a promising tumor characteristic marker in the future.

## CAF-Derived Exosomes in Cancer Progression

**Figure 3:**
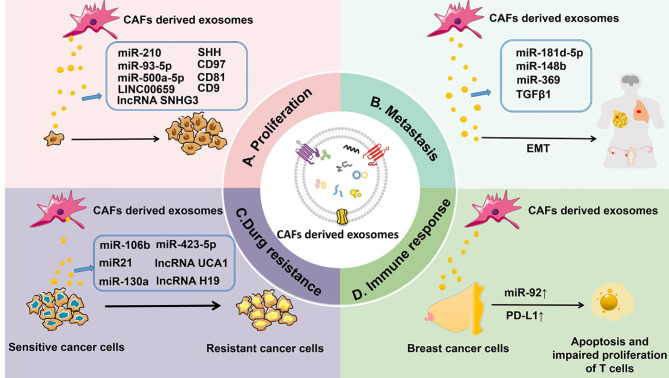
CAF-derived exosomes can regulate tumorigenesis. **(A)** CAF-derived exosomal ncRNAs (such as miR-210, miR-93-5p, miR-500a-5p, and lncRNAs LINC00659 and SNHG3) or proteins (such as SHH, CD97, CD81, and CD9) affect the proliferation of tumors. **(B)** CAF-derived exosomal ncRNAs (such as miR-181d-5p, miR-148b, and miR-369) or proteins (TGF-β) control the metastasis of tumors. **(C)** CAF-derived exosomal ncRNAs (such as miR-106b, miR-423-5p, miR-21, miR-130a, and lncRNAs UCA1 and H19) or proteins (TGF-β) influence drug resistance of tumors. **(D)** CAF-derived exosomal miR-92 and PD-L1 regulate the immune response to tumors by inducing apoptosis and impairing proliferation of T cells.

Lately, more and more studies indicate that CAF-derived exosomes play a crucial role in tumorigenesis, including tumor cell proliferation, metastasis, drug resistance, and immune responses (**Figure 3**).

### Tumor Proliferation

The proliferation of cancer cells plays a seminal part in tumor proliferation. Altered control of cell proliferation is the primary phenotypic feature of a malignant neoplastic cell population (122).

Various substances secreted by CAF-derived exosomes have been shown to be crucial for tumor cell proliferation. Among them, CAF-derived exosomal miRNAs are currently the focus of attention. For instance, in non–small cell lung carcinoma (NSCLC), CAF-derived exosomal miR-210 can promote EMT by targeting UPF1 and activating the PTEN/PI3K/AKT pathway and therefore can accelerate the growth of NSCLC (20). Chen et al. (123) have found that miR-93-5p contained in CAF-derived exosomes can enhance tumor growth in irradiated nude mice possibly by downregulating FOXA1 and upregulating TGFB3. In breast cancer, the proliferation of cancer cells is significantly accelerated after miR-500a-5p is transferred through CAF-derived exosomes (124). Aside from promoting tumor cell proliferation, CAF-derived exosomes can also inhibit tumor cell proliferation. Li et al. have demonstrated that exosomal miR-34a-5p was transferred from fibroblasts to oral squamous cell carcinoma cells and can bind to its direct downstream target AXL to suppress oral squamous cell carcinoma cell proliferation (125). Similarly, miR-3188 can be transferred from fibroblasts to head and neck cancer cells by exosomes and can influence the proliferation and apoptosis of head and neck cancer cells by directly targeting B-cell lymphoma 2 *in vitro* and *in vivo* (126). After being taken up by gastric cancer cells, gastric cancer fibroblast–derived exosomal miR-34 can inhibit gastric cancer cell proliferation (127). Investigation into the role of CAF-derived lncRNA is in progress. For example, CAF-derived exosomal lncRNA SNHG3 serves as a molecular sponge of miR-330-5p in breast cancer cells, which can enhance breast tumor cell proliferation (128). In CRC, LINC00659 transferred by CAF-derived exosomes directly interacts with miR-342-3p to increase the expression of ANXA2 in CRC cells, thereby promoting the proliferation of cancer cells (129). The role of CAF-derived exosomal proteins in tumor cell proliferation has also attracted much attention. As mentioned above, SHH-enriched exosomes secreted by CAFs can accelerate the growth and progression of ESCC by activating the SHH signaling pathway (60). Exosomal CD97 is responsible for mediating cancer cell proliferation through the mitogen-activated protein kinase pathway, as confirmed in a study of gastric cancers (130).

### Tumor Metastasis

Metastasis is the process by which tumor cells disseminate to distant tissues and adapt and survive in a foreign microenvironment (131, 132). The process of metastasis mainly includes several stages—local invasion, intravasation, survival in the circulation, extravasation, and finally colonization of a new site (133). Accumulating evidence shows that CAF-derived exosomes have a broad impact on tumor metastasis (114, 134, 135).

Metastasis requires the invasion of the primary tumor to break through the basement membrane and then to enter the circulation, and epithelial cells at the invasive front of carcinoma surmount this physical barrier by acquiring migratory and invasive properties through EMT (136). Accordingly, EMT of recipient cells is necessary for tumor invasion and metastasis, which can be enhanced by CAF-derived exosomes. Hu et al. (25) have demonstrated that CRC cells treated with CAF-derived exosomes overexpress mesenchymal markers (N-cadherin and vimentin) and underexpress epithelial markers (E-cadherin), suggesting that CAF-derived exsomes can induce EMT in CRC cells. Li et al. (101) have also found that TGF-β1 in CAF-derived exosomes can be transferred to ovarian cancer cells and promote EMT *via* the Smad signaling pathway, thereby contributing to metastasis. In breast cancer, by promoting the EMT of cancer cells *via* CDX2 and HOXA5, CAF-derived exosomes containing miR-181d-5p can enhance tumor metastasis and invasion (137). In endometrial cancer, CAF-derived exosomal miR-148b can be transferred to cancer cells and functions as a tumor suppressor by directly binding to its downstream target gene, DNMT1, to repress tumor metastasis by inducing EMT (21). Similarly, miR-369 promotes lung squamous cell carcinoma metastasis *in vivo* by inducing EMT (138).

Furthermore, CAF-derived exosomes may influence the metastasis of some tumors by establishing a premetastatic niche (PMN), which is an early event in cancer. Circulating tumor cells form a PMN before real metastasis to improve the chances of successful survival and settlement in a foreign microenvironment (139, 140). In salivary adenoid cystic carcinoma, CAF-derived EVs can induce lung PMN formation in mice and consequently increase lung metastasis of salivary adenoid cystic carcinoma; this process is associated the upregulation of plasma integrin β1 (141). It can also be hypothesized that as a significant type of EVs, CAF-derived exosomes may have the potential to form a PMN. However, only limited data are available on the ability of CAF-derived exosomes to stimulate the formation of a PMN. Therefore, the role of CAF-derived exosomes in PMN formation is unclear.

### Tumor Drug Resistance

Various methods can be used for cancer treatment, such as chemotherapy, drug therapy, and immunotherapy. Nonetheless, some types of cancer remain insensitive to these traditionally adjuvant treatments. The use of CAF-derived exosomes may be a promising strategy for overcoming a tumor’s treatment resistance.

CAF-derived exosomes mainly induce drug resistance by transferring miRNAs to adjacent cancer cells. For instance, Fang et al. have found that CAF-derived exosomes upregulate miR-106b; they confirmed that after direct transfer from CAFs to pancreatic cancer cells through exosomes, miR-106b can promote gemcitabine resistance of cancer cells by targeting TP53INP1 (142). In ovarian cancer, miR-21 can be transferred from CAFs to ovarian cancer cells, where it suppresses apoptosis and confers chemoresistance by binding to its recently discovered direct target, *APAF1* (143). Zhang et al. (8) have demonstrated that CAF-derived exosomes can confer cisplatin resistance upon NSCLC cells by transferring miR-130a. Prostate-cancer-associated-fibroblast–derived exosomes carrying miR-423-5p are reported to increase taxane resistance in prostate cancer through the TGF-β signaling pathway by targeting GREM2 (144). Just as miRNAs, CAF-derived exosomal lncRNAs are implicated in increased chemoresistance. Gao et al. (145) have shown that, as an miR-103a sponge, lncRNA UCA1 can confer resistance to cisplatin upon vulvar squamous cell carcinoma cells *in vitro* and *in vivo* through the miR-103a–WEE1 axis. Similarly, in CRC, lncRNA H19 functions as a competing endogenous RNA of miR-141 and contributes to the stemness of cancer stem cells, in this way leading to the activation of the Wnt–β-catenin signaling pathway. The transmission of exosomal H19 from CAFs to neighboring cells may be closely related to oxaliplatin resistance (115).

### Tumor Immune Response

The ability of tumors to escape surveillance by the immune system has long been considered an obstacle to the success of cancer immunotherapy. In recent years, immunotherapy emerged as a major breakthrough in cancer treatment. As described above, CAF-derived exosomes play a significant part in tumorigenesis, including tumor cell proliferation, metastasis, and drug resistance. Studies on the involvement of CAF-derived exosomes in tumor immune response are being actively conducted at present.

Immune cells include T cells, regulatory T cells, B cells, dendritic cells, natural killer cells, and others. We propose that the role of CAF-derived exosomes in the tumor immune response might be achieved by interacting with these immune cells. For example, in an experiment on cultured and isolated human breast CAF-derived exosomes, Dou et al. revealed that after treatment with CAF-derived exosomes, breast cancer cells overexpress PD-L1, accompanied by higher miR-92 levels, significantly promoting apoptosis and impairing the proliferation of T cells. This finding uncovered a novel mechanism to induce immune suppression in the TME (22).

So far, the effects of CAF-derived exosomes on immune cells in the TME have not been extensively studied, but on the basis of the role of CAFs in tumor immune response and the relation between CAFs and exosomes, we speculate that the participation of CAF-derived exosomes in tumor immune response is a promising research field, which would provide new targets for tumor prognostic indications and therapeutic strategies in the future.

## Clinic Value And Application of CAF-Derived Exosomes

### CAF-Derived Exosomes as Diagnostic Biomarkers

Cancer is a public health problem worldwide that is yet to be alleviated. In recent years, investigators revealed that aberrant levels of CAF-derived exosomal substances such as ncRNAs and proteins are closely associated with clinical pathological characterizations of cancer patients, including tumor–node–metastasis stage, lymph node metastasis status, and patient prognosis. Accumulating evidence indicates that various CAF-derived exosomal ncRNAs can be considered useful tumor-related biomarkers. Therefore, an increasing number of researchers are trying to devise breakthrough cancer treatments and to elucidate patients’ prognostic indicators.

To better understand the relation between CAF-derived exosomal contents and cancer, we provide a summary table (**Table 3**) with the aim of proposing new promising areas for the research on CAF-derived exosomes in cancer.

**Table 3:** Correlation between CAF-derived exosomal content and clinical pathological characterizations of cancer patients.

Exosomal contents	Tumor type	Sample sources	Dyregulation	Relationship with clinicopathology	Ref.
miR-382-5p	Oral squamous cell carcinoma	Tissue	Upregulation	TNM stage, lymph node metastasis	(135)
miR196a	Head and neck cancer	Plasma	Upregulation	Poor prognosis	(146)
miR-3188	Head and neck cancer	Plasma and tissue	Downregulation	TNM stage, tumor size, poor survival	(126)
miR-150-3p	Hepatocellular carcinoma	Plasma	Downregulation	Long survival	(61)
miR369	Lung squamous cell carcinoma	Tissue	Upregulation	Poor prognosis	(138)
miR-17-5p	Colorectal cancer	Tissue	Upregulation	Poor prognosis	(113)
lncRNA H19	Colorectal cancer	Tissue	Upregulation	TNM stage	(115)
lncRNA UCA1	Vulvar squamous cell carcinoma	Tissue	Upregulation	T stage, Clinical stage, and lymph node metastasis status	(145)
MMP11	Gastric cancer	Tissue	Upregulation	Poor prognosis	(47)

### CAF-Derived Exosomes as Therapeutic Target

**Figure 4:**
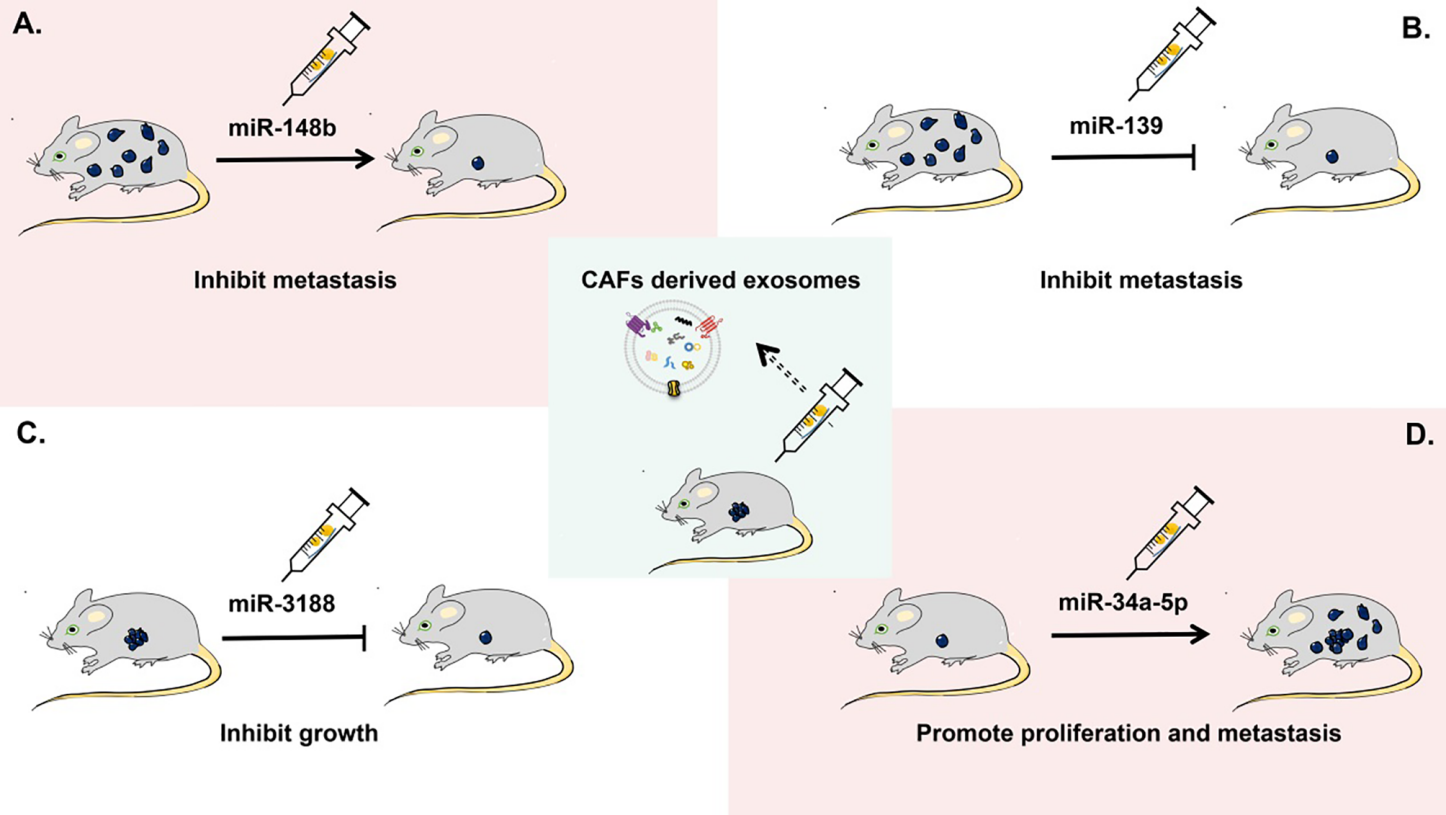
Applications of CAF-derived exosomes as therapeutic targets in cancer *in vivo*. In a mouse xenograft model, **(A)** CAF-derived exosomal miR-148b can inhibit the metastasis of endometrial cancer cells. **(B)** CAF-derived exosomal miR-139 can suppress the metastasis of gastric cancer cells. **(C)** CAF-derived exosomal miR-3188 is able to slow down the growth of head and neck squamous cell carcinoma cells. **(D)** CAF-derived exosomal miR-34a-5p can promote the proliferation and metastasis of oral squamous cell carcinoma cells.

CAF-derived exosomes have been proven to be helpful in predicting the prognosis of patients with tumor and providing potential targets for cancer treatment, but their clinical applications are currently limited. Recent studies on the role of CAF-derived exosomes in tumorigenesis are mainly at the stage of animal experiments (**Figure 4**). For example, in a xenograft nude-mouse model of head and neck cancer, injection of CAF-derived exosomal miR-3188 resulted in a smaller tumor burden (126). In a mouse model of gastric cancer, exosomal miR-139 has been shown to inhibit the metastasis of gastric cancer cells (47). A report on a nude-mouse model indicates that tumorigenicity including proliferation and metastasis of oral squamous cell carcinoma cells is significantly enhanced by the delivery of CAF-derived exosomal miR-34a-5p into cancer cells (125). In a nude-mouse model of lung metastasis from endometrial cancer cells, the metastasis is strongly reduced by overexpression of exosomal miR-148b in CAFs (21).

## Future Perspectives

CAFs, a key component of the TME, have received increasing attention in recent years. Exosomes can be regarded as mediators of information exchange in the TME mainly because of their biologically active contents, including ncRNAs, proteins, and metabolites. Clarifying the role of CAF-derived exosomes—i.e., newly studied EVs with special biological characteristics believed to be essential for tumorigenesis regulation—is important for the identification of novel diagnostic biomarkers and therapeutic targets in human cancers.

Previous studies on tumors have mainly focused on the study of cancer cells. Lately, aside from cancer cells, researchers have been focusing on the relation between the TME and tumorigenesis. An increasing number of studies have indicated that as a key mediator in the TME, CAF-derived exosomes play crucial roles in cancer initiation and progression. CAF-derived exosomes are EVs with distinct biological characteristics. First, exosomes can be considered nanocarriers (i.e., carriers with very small size) and have already been tested as nanomaterials for drug delivery. Because of the membrane-based structure of exosomes, their contents can be better protected from degradation. This property makes CAF-derived exosomes a crucial carrier of biological substances for information exchange in the TME. Second, exosomes are a type of EVs present in all types of body fluids with good biocompatibility and circulatory stability. Therefore, when CAF-derived exosomes are used in clinical treatment, we speculate that they can stably exist in the targeted site and cause no immunological rejection *in vivo*. Third, CAFs are key components of the TME. After secretion by CAFs into the TME, exosomes containing bioactive factors such as ncRNAs, proteins, and some metabolites perform indispensable roles in oncogenesis and cancer progression. They can be taken up by surrounding recipient cells and trigger a series of responses inside these recipient cells, including regulating signaling pathways and targeting specific genes. In this way, CAF-derived exosomes can significantly affect tumor cell proliferation, metastasis, drug resistance, and immune responses. According to the existing data, we suggest that the role of CAF-derived exosomes in tumorigenesis is a promising research field for updating tumor therapeutic strategies (21, 22, 129, 147).

However, there are still some problems in the study of CAF-derived exosomes. First, the methods for separation and purification of exosomes vary, and there is no recognized effective extraction technique for obtaining high-purity exosomes; this situation means difficulties with the study of CAF-derived exosomes. Second, the TME is extremely complex. The specific mechanism of CAF-derived exosomes on their recipient tumor cells that affects malignant tumor behaviors needs to be further elucidated. Third, in terms of translational research and clinical applications, there are not enough animal experiments verifying the effectiveness of CAF-derived exosomes in the treatment of cancers, and there are few clinical studies. Although exosomes have been adapted to serve as nanomaterials carrying drugs, there is little evidence that they can stably maintain an ideal functional state of a drug *in vivo*.

With additional in-depth and innovative research, we believe that the role of CAF-derived exosomes in tumorigenesis will be clarified further, and more encouraging progress will be made soon.

## Author Contributions

CO and JW contributed to the conception and design of the study. LP performed resource analysis, and wrote the first draft of the manuscript. All authors contributed to manuscript revision and read and approved the submitted version.

## Funding

This work was supported by the National Natural Science Foundation of China (No. 81602167 and 81903032), the China Postdoctoral Science Foundation (No. 2020M672520), the Hunan Provincial Natural Science Foundation of China (No. 2021JJ31100 and 2021JJ4101), the Science and Technology Program Foundation of Changsha City (No. kq2004085) and the Youth Fund of Xiangya Hospital (2018Q011).

## Conflict of Interest

The authors declare that the research was conducted in the absence of any commercial or financial relationships that could be construed as a potential conflict of interest.

## Publisher’s Note

All claims expressed in this article are solely those of the authors and do not necessarily represent those of their affiliated organizations, or those of the publisher, the editors and the reviewers. Any product that may be evaluated in this article, or claim that may be made by its manufacturer, is not guaranteed or endorsed by the publisher.

## Abbreviations

CAFs, cancer-associated fibroblasts; circRNA, circular RNA; CRC, colorectal cancer; ECM, extracellular matrix; EMT, epithelial–mesenchymal transition; ESCC, esophageal squamous cell carcinoma; EVs, extracellular vesicles; HCC, hepatocellular carcinoma; HSP, heat shock protein; IL-6, interleukin 6; lncRNA, long noncoding RNA; miR; miRNA, microRNA; mRNA, messenger RNA; MVB, multivesicular body; ncRNA, noncoding RNA; NFs, normal fibroblasts; NSCLC, non–small cell lung cancer; PD-L1, programmed death ligand 1; PMN, premetastatic niche; SHH, sonic hedgehog; TGF-β, transforming growth factor β; TME, tumor microenvironment.
